# Adrenergic gene polymorphisms and cardiovascular risk in the NHLBI-sponsored Women's Ischemia Syndrome Evaluation

**DOI:** 10.1186/1479-5876-6-11

**Published:** 2008-03-10

**Authors:** Michael A Pacanowski, Issam Zineh, Haihong Li, B Delia Johnson, Rhonda M Cooper-DeHoff, Vera Bittner, Dennis M McNamara, Barry L Sharaf, C Noel Bairey Merz, Carl J Pepine, Julie A Johnson

**Affiliations:** 1 Department of Pharmacy Practice and Center for Pharmacogenomics, University of Florida College of Pharmacy, Gainesville, FL, USA; 2 Department of Epidemiology and Health Policy Research, University of Florida College of Medicine, Gainesville, FL, USA; 3 Department of Epidemiology, University of Pittsburgh Graduate School of Public Health, Pittsburgh, PA, USA; 4 Department of Medicine, University of Florida College of Medicine, Gainesville, FL, USA; 5 Department of Medicine, University of Alabama Birmingham, Birmingham, AL, USA; 6 Department of Medicine, University of Pittsburgh School of Medicine, Pittsburgh, PA, USA; 7 Department of Medicine, Rhode Island Hospital, Providence, RI, USA; 8 Department of Medicine and Cedars-Sinai Research Institute, Cedars-Sinai Medical Center, Los Angeles, CA, USA

## Abstract

**Background:**

Adrenergic gene polymorphisms are associated with cardiovascular and metabolic phenotypes. We investigated the influence of adrenergic gene polymorphisms on cardiovascular risk in women with suspected myocardial ischemia.

**Methods:**

We genotyped 628 women referred for coronary angiography for eight polymorphisms in the α_1A_-, β_1_-, β_2_- and β_3_-adrenergic receptors (*ADRA1A*, *ADRB1, ADRB2*, *ADRB3*, respectively), and their signaling proteins, G-protein β 3 subunit (*GNB3*) and G-protein α subunit (*GNAS*). We compared the incidence of death, myocardial infarction, stroke, or heart failure between genotype groups in all women and women without obstructive coronary stenoses.

**Results:**

After a median of 5.8 years of follow-up, 115 women had an event. Patients with the *ADRB1 *Gly389 polymorphism were at higher risk for the composite outcome due to higher rates of myocardial infarction (adjusted hazard ratio [HR] 3.63, 95% confidence interval [95%CI] 1.17–11.28; Gly/Gly vs. Arg/Arg HR 4.14, 95%CI 0.88–19.6). The risk associated with *ADRB1 *Gly389 was limited to those without obstructive CAD (n = 400, P_interaction _= 0.03), albeit marginally significant in this subset (HR 1.71, 95%CI 0.91–3.19). Additionally, women without obstructive CAD carrying the *ADRB3 *Arg64 variant were at higher risk for the composite endpoint (HR 2.10, 95%CI 1.05–4.24) due to subtle increases in risk for all of the individual endpoints. No genetic associations were present in women with obstructive CAD.

**Conclusion:**

In this exploratory analysis, common coding polymorphisms in the β_1_- and β_3_-adrenergic receptors increased cardiovascular risk in women referred for diagnostic angiography, and could improve risk assessment, particularly for women without evidence of obstructive CAD.

**Trial Registration:**

ClinicalTrials.gov NCT00000554.

## Background

Coronary artery disease (CAD) is the leading cause of morbidity and mortality among women in the United States [1]. More than half of women presenting with chest pain or suspected myocardial ischemia do not have angiographic evidence of stenosis [2]. Despite the absence of obstructive lesions, many of these women have been shown to have myocardial ischemia due to microvascular disease [3,4] and are at high risk for cardiovascular events [5,6]. Diagnosing CAD and assessing cardiovascular risk among women continues to be clinically challenging and represents a major public health concern. Therefore, alternative methods to estimate cardiovascular risk in women are necessary to reduce the burden of cardiovascular disease.

Cardiovascular disease has been observed in families, and a genetic predisposition has long been appreciated [7]. The literature is replete with studies have demonstrated the potential prognostic value of genetic polymorphisms [8], even in patients with established cardiovascular disease [9-11]. Studies have also demonstrated a sex-specific associations between genetic variants and cardiovascular disease phenotypes such as myocardial infarction and ischemic heart disease [12]. However, the potential genetic mechanisms remain incompletely explored.

Genetic polymorphisms in the adrenergic system have been linked to various cardiovascular and metabolic disorders, such as hypertension, heart failure, and diabetes [13] Namely, the genes that encode the β_1_-, β_2_-, and α_1_-adrenergic receptors are important in myocardial and vascular function, the β_3_-adrenergic receptors are involved in thermogenesis and lipolysis, and the subunits of their cognate G proteins all have documented associations with cardiovascular or metabolic phenotypes. We investigated the association of these genes with cardiovascular outcomes in women with clinical indications for a cardiac angiography who participated in the National Heart, Lung, and Blood Institute (NHLBI)-sponsored Women's Ischemia Syndrome Evaluation (WISE) study.

## Methods

### Study population and procedures

We studied 628 women enrolled in the NHLBI-sponsored WISE study who consented to genetic analyses and had complete clinical data. The WISE protocol has been previously described [14]. Briefly, the WISE was a multicenter prospective cohort study of 936 women that was designed to evaluate diagnostic techniques, disease mechanisms, and the prognosis of ischemic heart disease in women, particularly those without coronary artery stenosis. The WISE population consisted of women over the age of 18 undergoing coronary angiography as clinically indicated for the evaluation of chest pain or suspected myocardial ischemia. The baseline evaluation included collection of demographic data and a detailed medical history, as well as a symptom and psychosocial evaluation, physical examination, and blood sampling. Quantitative angiography was performed at a core laboratory by investigators blinded to all other subject data. Follow-up data were collected by telephone or mail contact six weeks after angiography, then yearly. Women were followed for death from any cause and hospitalization for nonfatal myocardial infarction (MI), heart failure, or stroke. Death certificates were obtained for verification and, where possible, other events were verified against the medical record. Nonfatal events were adjudicated at one center and shown to be 98.2% concordant with data gathered through standard follow-up procedures. The WISE protocol was approved by the institutional review boards of all participating sites, and all study participants gave written informed consent before undergoing evaluation and sample collection for genetic analyses.

### Selection of polymorphisms and genotyping methods

Genomic DNA was isolated from whole blood using a commercially available kit (Puregene; Gentra Systems, Minneapolis, MN). Genotypes were determined for the following 8 single nucleotide polymorphisms (SNPs) with known or putative functional consequences in 6 adrenergic system genes: β_1_-adrenergic receptor (*ADRB1*; Ser49Gly and Arg389Gly), β_2_-adrenergic receptor (*ADRB2*; Arg16Gly and Gln27Glu), β_3_-adrenergic receptor (*ADRB3*; Trp64Arg), α_1A_-adrenergic receptor (*ADRA1A*; Arg347Cys), stimulatory G-protein α subunit (*GNAS*; 393 T>C), and G-protein β3 subunit (*GNB3*; 825 C>T). Genotyping was performed in duplicate using optimized polymerase chain reaction protocols and either single-primer extension (SNP-IT; Orchid Biosciences, Princeton, NJ) or luciferase-based assays with the Pyrosequencing PSQ HS 96 system (Biotage AB, Uppsala, Sweden).

### Data analysis

Baseline characteristics were compared between genotype groups using χ^2 ^tests for categorical data and t-tests, analysis of variance, or a nonparametric equivalent for continuous data. Departures from Hardy-Weinberg equilibrium were tested by χ^2 ^or Fisher's exact tests. The primary outcome was a composite of death from any cause, or hospitalization for nonfatal MI, heart failure, or stroke. The effect of each of the eight SNPs on the primary outcome was evaluated using Kaplan-Meier analysis and pooled log-rank tests adjusted for race. Hazard ratios (HR) and 95% confidence intervals (95%CI) were estimated using Cox proportional hazards regression. The regression model included age and race as forced covariates, in addition to clinical variables that were significant predictors of the outcome in univariate analysis (P < 0.1) that remained significant in multivariate analysis (P < 0.05). Given the heterogeneous nature of the primary outcome, significant associations were followed by exploratory analysis of the individual outcomes. All analyses compared heterozygotes and variant homozygotes to common allele homozygotes, except SNPs with a minor allele frequency < 0.1 were treated as dominant in the interest of ensuring adequate power. Furthermore, where more than 1 SNP was typed in a single gene (i.e. *ADRB1 *and *ADRB2*), haplotypes were inferred using PHASE version 2.1 [15], coded based on the number of copies (0, 1, or 2), and individually entered into the regression with 0 copies as the referent category. To probe for modification of genotype effects by race and CAD severity, interaction terms were tested and stratified analysis was performed.

Logic regression under the Cox proportional hazards model, implemented as a package for R statistical software, was used to define epistatic interactions as previously described [16-18]. Logic regression is a powerful adaptive regression method that tests multiple variable combinations (leaves) using logical, Boolean operators (and, or) and stochastic modeling to define the best fitting model (tree) for a binary outcome. As an example, this method yields a model that is interpreted as follows: (SNP A carrier AND SNP B variant homozygote) OR ((SNP C carrier OR SNP D carrier) AND SNP E carrier). Cross-validation was performed to identify the best scoring model containing 1 to 8 predictors, and a single logic regression tree was constructed. Default settings were used with 25,000 iterations. All SNPs were eligible for inclusion in the model. Additional models were fit for women without obstructive CAD.

An additional analysis was performed to determine if genetic markers retained predictive value along with clinical data and inflammatory biomarkers for women with available data (n = 559, 89%). This model added global inflammatory status, as defined by the number of inflammatory biomarkers in the upper quartile (C-reactive protein, interleukin-6, and serum amyloid A) [19]. The contribution of genetic variables to the overall risk assessment beyond clinical and inflammatory variables among women without obstructive CAD was determined by comparing the global -2 log-likelihood of the models containing clinical and inflammatory biomarker data, with and without significant genetic variables.

Statistical analyses were performed using SAS version 9.1 (SAS Institute, Inc., Cary, NC) and R (R Foundation, Vienna, Austria). The significance threshold for all analyses was set at α = 0.05. At minor allele frequencies greater than 0.1, this study had 80% power to detect a relative hazard of approximately 1.81 assuming a standard deviation of 0.5 and a dominant model. Power calculations were performed using PASS (NCSS, Kaysville, UT).

## Results

Complete clinical and genetic data were available for 628 women. Baseline characteristics are presented in Table 1. The majority of women were non-Hispanic whites (83%). The mean age was 58 ± 12 years and 32% of the women were over the age of 65. Obstructive lesions on angiography were present in 228 (36%) patients. Hardy-Weinberg equilibrium was satisfied for all loci. Genotype and allele frequencies differed significantly by race at all loci (Table 2). Notable differences in baseline characteristics by genotype were as follows: *ADRA1A *Cys347 homozygotes had a lower prevalence of dyslipidemia (Arg/Arg 53%, Arg/Cys 50%, Cys/Cys 40%, P = 0.04) and higher diastolic blood pressure (Arg/Arg 75 ± 10 mmHg Arg/Cys 77 ± 11 mmHg, Cys/Cys 79 ± 11 mmHg, P = 0.02), *GNAS *393C homozygotes had a lower hypertension prevalence (T/T 65% T/C 58% C/C 52%, P = 0.05) and systolic BP (139 ± 23 mmHg, 137 ± 21 mmHg, 133 ± 19 mmHg, P = 0.02), and *GNB3 *825T homozygotes had a higher prevalence of diabetes (C/C 21%, C/T 20%, T/T 40%, P = 0.0002).

**Table 1:** Baseline characteristics

**Characteristic**	≥ **50% stenosis (n = 228)**	** < 50% stenosis (n = 400)**
White/Caucasian (%)	81	85
Age (years)*	62 ± 12	56 ± 11
Past medical history (%)		
Diabetes mellitus*	38	16
Hypertension*	67	54
Dyslipidemia*	63	40
Systolic blood pressure (mmHg)	138 ± 22	136 ± 21
Diastolic blood pressure (mmHg)	77 ± 11	77 ± 11
Body mass index (kg/m^2^)	29.1 ± 6.4	29.8 ± 6.8
Waist circumference (in)	36 ± 8	36 ± 7
Cigarette smoking (%)		
Currently	22	19
Past	37	33
Menopausal status (%)*		
Pre-menopausal	13	19
Peri-menopausal	4	10
Post-menopausal	82	70
Family history of premature CAD (%)	65	66
Current medication use (%)		
Aspirin*	75	49
Statin*	37	22
ACE-inhibitor*	29	22
β-blocker*	50	34
History of HRT (%)*	40	54
CRP (n = 547; median, IQR)	0.37, 0.17–0.97	0.36, 0.16–0.84
SAA (n = 519; median, IQR)*	0.66, 0.36–1.15	0.55, 0.31–0.94
IL-6 (n = 547; median, IQR)	3.37, 1.85–6.87	2.79, 1.72–4.61

Abbreviations: ACE, angiotensin-converting enzyme; CAD, coronary artery disease; HRT, hormone replacement therapy; IQR, interquartile range; CRP, C-reactive protein; SAA, serum amyloid A; IL-6, interleukin-6

Values presented as means+SD unless otherwise specified

*P < 0.05 for comparison between > 50% stenosis and < 50% stenosis; for CRP, SAA, and IL-6 based on Wilcoxon rank-sum test

^†^History of high cholesterol or use of lipid-lowering medication

**Table 2:** Genotype and allele frequencies by race*

**Gene**	**Polymorphism**	**Genotype**	**White, n (%)**	**Black, n (%)**
*ADRB1*	Ser49Gly	Ser/Ser	399 (76)	49 (47)
		Ser/Gly	121 (23)	49 (47)
		Gly/Gly	3 (1)	6 (6)
		MAF	0.12	0.29
	Arg389Gly	Arg/Arg	265 (51)	37 (36)
		Arg/Gly	215 (41)	54 (52)
		Gly/Gly	41 (8)	13 (12)
		MAF	0.29	0.38
*ADRB2*	Gly16Arg	Gly/Gly	175 (33)	18 (17)
		Gly/Arg	263 (50)	56 (54)
		Arg/Arg	86 (16)	30 (29)
		MAF	0.42	0.56
	Gln27Glu	Gln/Gln	193 (37)	74 (71)
		Gln/Glu	253 (48)	29 (28)
		Glu/Glu	77 (15)	1 (1)
		MAF	0.39	0.15
*ADRB3*	Trp64Arg	Trp/Trp	439 (84)	81 (79)
		Trp/Arg	78 (15)	21 (20)
		Arg/Arg	3 (1)	1 (1)
		MAF	0.08	0.11
*ADRA1A*	Arg347Cys	Arg/Arg	163 (31)	6 (6)
		Arg/Cys	270 (52)	43 (41)
		Cys/Cys	91 (17)	55 (53)
		MAF	0.43	0.74
*GNAS*	393 T > C	T/T	139 (27)	60 (58)
		T/C	254 (49)	42 (40)
		C/C	130 (25)	2 (2)
		MAF	0.49	0.22
*GNB3*	825 C > T	C/C	280 (53)	7 (7)
		C/T	210 (40)	30 (29)
		T/T	34 (7)	67 (64)
		MAF	0.27	0.79

Abbreviations: MAF, minor allele frequency

*all P < 0.05 for genotype frequency by race

The median duration of follow-up was 5.8 years (interquartile range 3.6–8.1 years), over which time 115 women (18.3%) experienced a primary event. The event rate was higher in women with (versus without) obstructive CAD (28.9% vs. 12.3%). Clinical correlates with event risk identified in univariate analysis included baseline systolic BP, age, black race, ever-smoking, diabetes history, hypertension history, dyslipidemia history, BMI, and obstructive CAD. Hypertension and dyslipidemia history did not retain significance in the multivariate model and were eliminated. Additionally, the risk for the composite outcome was higher among patients with 2 or more inflammatory markers in the highest quartile, as previously reported [19]. All of these factors were predictive in patients without obstructive CAD, whereas only diabetes, ever-smoking, and inflammatory biomarkers were associated with a greater risk for the primary outcome in women with obstructive CAD (data not shown).

Associations between the adrenergic SNPs and the primary outcome are depicted in Table 3. Patients with the *ADRB1 *Gly389 allele had a significantly higher incidence of the primary outcome (log-rank P = 0.047; Table 3). When restricted to whites only, the risk in *ADRB1 *Gly389 homozygotes was elevated but marginally significant (Table 3). The primary outcome association was driven by higher rates of MI among patients with the variant allele and death and heart failure trended in the same direction, while stroke risk was neutral (Table 4). For *ADRB1*, 3 common haplotypes were defined; the Gly49-Gly389 haplotype was not observed. Consistent with the SNP associations, patients with 2 copies of the Ser49-Gly389 haplotype were at increased risk for the composite outcome (frequency 0.30; HR 2.00, 95%CI 1.08–3.71), and similar trends were noted for the individual endpoints as above. No other significant SNP associations were identified in the overall population (Table 3). For *ADRB2*, 3 common haplotypes were defined; the Arg16-Gly27 allele was present on only 9 chromosomes in whites. *ADRB2 *haplotypes were not associated with the primary outcome (data not shown).

**Table 3:** Incidence and relative hazard of primary outcome by genotype

**Gene**	**Genotype**	**No. Events (%)**	Primary Outcome Incidence*	**All Patients Adjusted HR (95%CI)^† ^n = 628**	**P**	**Whites Adjusted HR (95%CI)^† ^n = 524**	**P**	**Blacks Adjusted HR (95%CI)^† ^n = 104**	**P**
***ADRB1***									
Ser49Gly	Ser/Ser	82 (18)	4.2	1.00 (reference)		1.00 (reference)		1.00 (reference)	
	Ser/Gly	30 (18)	3.8	0.88 (0.57–1.37)	0.58	0.89 (0.51–1.53)	0.66	1.12 (0.51–2.48)	0.78
	Gly/Gly	3 (33)	6.4	1.02 (0.31–3.36)	0.98	...	0.98	1.95 (0.47–8.03)	0.36
Arg389Gly	Arg/Arg	44 (15)	3.2	1.00 (reference)		1.00 (reference)		1.00 (reference)	
	Arg/Gly	55 (20)	4.5	1.15 (0.77–1.71)	0.49	1.09 (0.69–1.72)	0.71	1.23 (0.56–2.83)	0.62
	Gly/Gly	14 (26)	7.2	1.94 (1.05–3.59)	0.03	1.82 (0.92–3.61)	0.09	2.27 (0.53–9.79)	0.27
***ADRB2***									
Gly16Arg	Gly/Gly	34 (18)	4.0	1.00 (reference)		1.00 (reference)		1.00 (reference)	
	Gly/Arg	57 (18)	4.0	0.93 (0.60–1.43)	0.73	0.90 (0.56–1.46)	0.67	1.09 (0.38–3.19)	0.87
	Arg/Arg	24 (21)	4.6	1.14 (0.67–1.93)	0.63	0.81 (0.42–1.57)	0.53	2.61 (0.81–8.42)	0.11
Gln27Glu	Gln/Gln	52 (20)	4.4	1.00 (reference)					
	Gln/Glu	49 (17)	3.8	0.88 (0.59–1.30)	0.51	1.06 (0.67–1.71)	0.79	0.51 (0.20–1.30)	0.16
	Glu/Glu	14 (18)	4.4	0.99 (0.53–1.84)	0.98	1.13 (0.59–2.15)	0.72	...	0.99
***ADRB3***^‡^									
Trp64Arg	Trp/Trp	93 (18)	4.0	1.00 (reference)		1.00 (reference)		1.00 (reference)	
	Trp/Arg + Arg/Arg	21 (20)	4.8	1.32 (0.81–2.14)	0.26	1.19 (0.67–2.14)	0.55	1.33 (0.51–3.50)	0.56
***ADRA1A***									
Arg347Cys	Arg/Arg	31 (18)	4.1	1.00 (reference)		1.00 (reference)		1.00 (reference)	
	Arg/Cys	58 (19)	4.4	1.03 (0.66–1.62)	0.88	0.97 (0.60–1.57)	0.89	1.05 (0.20–5.43)	0.95
	Cys/Cys	26 (18)	3.5	0.68 (0.37–1.18)	0.16	0.83 (0.43–1.58)	0.56	0.46 (0.08–2.39)	0.35
***GNAS***									
393 T > C	T/T	36 (18)	3.9	1.00 (reference)		1.00 (reference)		1.00 (reference)	
	T/C	58 (20)	4.5	1.10 (0.71–1.69)	0.67	1.26 (0.73–2.17)	0.40	0.97 (0.39–1.93)	0.74
	C/C	21 (16)	3.6	1.31 (0.74–2.31)	0.36	1.60 (0.85–3.01)	0.15	...	0.99
***GNB3***									
825 C > T	C/C	47 (16)	3.7	1.00 (reference)		1.00 (reference)		1.00 (reference)	
	C/T	42 (18)	3.8	1.06 (0.69–1.64)	0.78	1.26 (0.73–2.17)	0.40	0.87 (0.40–1.93)	0.74
	T/T	26 (26)	6.1	1.51 (0.82–2.77)	0.19	1.60 (0.85–3.01)	0.15	...	0.99

Abbreviations: HR, hazard ratio; 95%CI, 95% confidence interval

* incidence per 100 patient-years

^† ^adjusted for age, race, presence of obstructive CAD, history of diabetes, ever-smoking, baseline systolic BP, BMI

^‡ ^analyzed using dominant model based on minor allele frequency ≤ 0.1

**Table 4:** Individual endpoint risk by *ADRB1 *and *ADRB3 *genotype

	**Death**		**Myocardial Infarction**		**Heart Failure**		**Stroke**	
	**No. Events (%)**	Adjusted HR (95%CI)* **n = 628**	**No. Events (%)**	Adjusted HR (95%CI)* **n = 628**	**No. Events (%)**	Adjusted HR (95%CI)* **n = 628**	**No. Events (%)**	Adjusted HR (95%CI)* **n = 628**

**All Patients (n = 628)**								
***ADRB1 *Arg389Gly**								
Arg/Arg	22 (7.3)	1.00 (reference)	4 (1.3)	1.00 (reference)	11 (3.6)	1.00 (reference)	14 (4.6)	1.00 (reference)
Arg/Gly	29 (10.8)	1.27 (0.73–2.23)	13 (4.8)	3.35 (1.08–10.3)	16 (6.0)	1.28 (0.58–2.80)	9 (3.4)	0.61 (0.26–1.41)
Gly/Gly	7 (13.0)	1.65 (0.68–3.99)	3 (5.6)	3.89 (0.84–18.1)	4 (7.4)	2.12 (0.65–6.91)	4 (7.4)	1.53 (0.49–4.77)
***ADRB3 *Trp64Arg**^†^								
Trp/Trp	50 (9.6)	1.00 (reference)	16 (3.1)	1.00 (reference)	24 (4.6)	1.00 (reference)	21 (4.0)	1.00 (reference)
Trp/Arg + Arg/Arg	10 (9.7)	1.01 (0.50–2.04)	4 (3.9)	1.47 (0.48–4.52)	6 (5.8)	1.46 (0.58–3.67)	5 (4.8)	1.20 (0.45–3.24)

**Non-obstructive CAD (n = 400)**								
***ADRB1***								
**Arg389Gly**								
Arg/Arg	8 (4.1)	1.00 (reference)	0	1.00 (reference)	3 (1.5)	1.00 (reference)	5 (2.6)	1.00 (reference)
Arg/Gly	8 (4.8)	0.77 (0.29–2.05)	8 (4.8)	...	11 (6.6)	4.37 (1.12–17.0)	6 (3.6)	1.06 (0.31–3.66)
Gly/Gly	4 (11.1)	2.08 (0.60–7.18)	2 (5.6)	...	0	...	2 (5.6)	1.54 (0.29–8.31)
***ADRB3***								
**Trp64Arg**^†^								
Trp/Trp	17 (4.9)	1.00 (reference)	7 (2.1)	1.00 (reference)	11 (3.2)	1.00 (reference)	10 (2.9)	1.00 (reference)
Trp/Arg + Arg/Arg	4 (6.9)	1.91 (0.61–5.97)	3 (5.2)	2.94 (0.69–12.5)	3 (5.2)	2.10 (0.55–8.10)	3 (5.2)	2.35 (0.60–9.29)

Abbreviations: HR, hazard ratio; 95%CI, 95% confidence interval

* adjusted for age, race, presence of obstructive CAD, history of diabetes, ever-smoking, baseline systolic BP, BMI

^† ^analyzed using dominant model based on minor allele frequency ≤ 0.1

**Figure 1:**
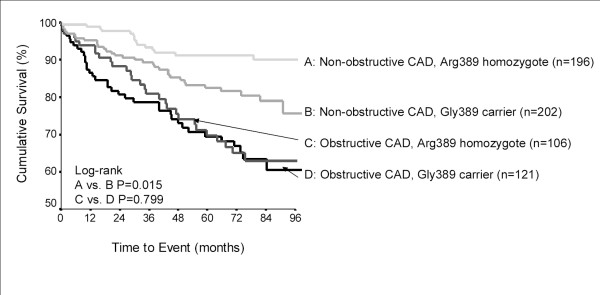
Kaplan-Meier plot for primary outcome by *ADRB1 *codon 389 genotype and CAD severity.

**Figure 2:**
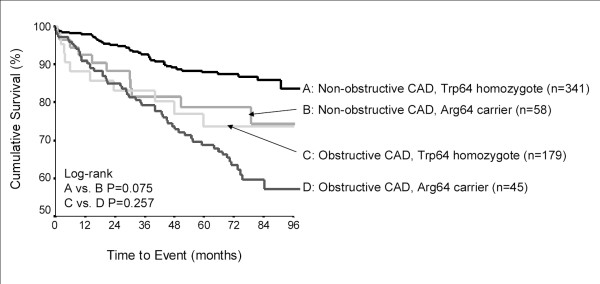
Kaplan-Meier plot for primary outcome by *ADRB3 *codon 64 genotype and CAD severity.

The presence or absence of CAD appeared to modify genotype risks for *ADRB1 *Arg389Gly (P_interaction _= 0.03; Figure 1) and *ADRB3 *Trp64Arg (P_interaction _= 0.1; Figure 2). None of the SNPs were associated with the primary outcome in patients with obstructive CAD (data not shown). However, in women without obstructive CAD, the association between *ADRB1 *Arg389Gly polymorphism and the primary outcome remained significant (log-rank P = 0.03) but was attenuated when adjusted for clinical covariates (Arg/Gly vs. Arg/Arg HR 1.56, 95%CI 0.82–2.97; Gly/Gly vs. Arg/Arg HR 1.95, 95%CI 0.82–2.97). Gly389 carriers were at increased risk in both racial groups (data not shown). Again, this was related to increased MI risk among Gly389 carriers, in addition to an increase in heart failure risk (Table 4). Haplotype associations were predominantly driven by patients with 2 copies of the Ser49-Gly389 haplotype (HR 1.74, 95%CI 0.52–1.50), but not statistically significant. Also in the subgroup of women without obstructive CAD, a trend toward higher risk was apparent in patients carrying the Arg64 allele of *ADRB3 *(log-rank P = 0.074). The association strengthened after adjustment for clinical covariates (Trp/Arg+Arg/Arg vs. Trp/Trp HR 2.10, 95%CI 1.05–4.24). The risk among Arg 64 carriers remained significant when restricted to whites (Trp/Arg+Arg/Arg vs. Trp/Trp HR 2.49, 95%CI 1.11–5.60). *ADRB3 *genotype was associated with subtle trends toward higher rates of all events (Table 4).

**Figure 3:**
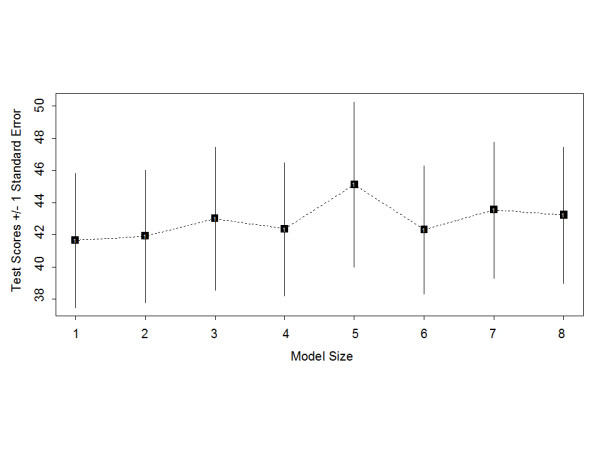
**Test-set deviance for different size logic regression models**. Test-set deviances from cross-validation analysis are shown for single logic regression trees containing 1 to 8 predictors. Lower test-scores represent the better-fitting model.

Logic regression models with 1 to 8 predictors were evaluated. Figure 3 illustrates the test-set deviance for different size models in the overall population. Models containing more than 1 predictor did not improve test-set deviance, and the best fitting model for the overall population and the subset of patients with obstructive CAD (not including other clinical predictors) contained only the *ADRB1 *Arg389Gly polymorphism.

Including inflammatory biomarkers in the model reduced the sample size by 11%. Consequently, while the increased risk for the composite outcome associated with the Gly389 persisted after adjustment for inflammatory biomarkers, the estimate lost significance (Arg/Gly vs. Arg/Arg HR 0.96, 95%CI 0.63–1.47; Gly/Gly vs. Arg/Arg HR 1.83, 95%CI 0.95–3.52). The association with MI was similarly borderline in the overall population (Arg/Gly vs. Arg/Arg HR 3.12, 95%CI 0.94–10.4; Gly/Gly vs. Arg/Arg HR 3.37, 95%CI 0.56–20.4) and when restricted to women without obstructive CAD (Arg/Gly vs. Arg/Arg HR 4.83, 95%CI 0.84–20.32; Gly/Gly vs. Arg/Arg not calculated because no events in Gly/Gly patients). However, despite the sample size reduction, the risk associated with carrying the *ADRB3 *Arg64 allele remained significant in women without obstructive CAD (Trp/Arg+Arg/Arg vs. Trp/Trp HR 2.37, 95%CI 1.08–5.20). For patients without obstructive CAD, incorporating both the *ADRB1 *codon 389 and *ADRB3 *codon 64 genotypes significantly improved the global -2 log-likelihood of the models based on clinical variables (P < 0.001 for change) and inflammatory biomarker data (P < 0.001 for change; Table 5).

**Table 5:** Multivariate Cox proportional hazards regression model for the primary outcome in women without obstructive CAD*

**Factor**	**HR (95%CI)**	**P**
***Clinical Model with Genetic Biomarkers *(n = 396)**		
*ADRB1 *Gly389 carrier	1.71 (0.91–3.19)	0.09
*ADRB3 *Arg64 carrier	2.14 (1.06–4.30)	0.03
Black race	1.77 (0.90–3.48)	0.10
Age (per decade)	1.02 (0.99–1.05)	0.25
History of diabetes	4.08 (2.01–8.28)	< 0.001
Ever-smoking	8.13 (3.55–18.63)	< 0.001
Baseline systolic BP (per 10 mmHg)	1.16 (0.99–1.36)	0.06
Body mass index (per 5 kg/m^2^)	0.70 (0.54–0.92)	0.01
		
***Clinical Model with Inflammatory and Genetic Biomarkers *(n = 355)**		
*ADRB1 *Gly389 carrier	1.67 (0.85–3.27)	0.13
*ADRB3 *Arg64 carrier	2.43 (1.11–5.35)	0.03
Black race	1.84 (0.92–3.67)	0.08
Age (per decade)	1.01 (0.97–1.04)	0.66
History of diabetes	3.78 (1.78–8.02)	< 0.001
Ever-smoking	7.43 (3.13–17.63)	< 0.001
Baseline systolic BP (per 10 mmHg)	1.17 (0.98–1.37)	0.08
Body mass index (per 5 kg/m^2^)	0.70 (0.53–0.93)	0.01
1 inflammatory biomarker in upper quartile*	1.67 (0.75–3.77)	0.21
2 or 3 inflammatory biomarkers in upper quartile	4.30 (2.02–9.16)	< 0.001

Abbreviations: HR, hazard ratio; 95%CI, 95% confidence interval*

* Hypertension and dyslipidemia eliminated from models after stepwise selection

## Discussion

In this study, we examined the joint effect of traditional risk factors, inflammatory mediators, and candidate SNPs in several genes from the adrenergic pathway on cardiovascular risk in women undergoing clinical evaluation for suspected ischemia. Women with symptoms of ischemia prompting angiography have been shown to have microvascular CAD, which is associated with a prognosis that is similar to that of patients with obstructive CAD [4,6]. We identified associations between incident cardiovascular events and the β_1_-adrenergic receptor Arg389Gly and the β_3_-adrenergic receptor Trp64Arg polymorphisms. The association with *ADRB3 *was present only in women without obstructive CAD and maintained significance in the presence of other robust predictors of cardiovascular risk.

The Arg389Gly polymorphism in *ADRB1 *was associated with nearly a two-fold increase in the risk for major cardiovascular events. *In vitro*, the Arg389 allele demonstrates higher basal and agonist-stimulated adenylyl cyclase activity than the Gly389 variant, resulting in increased sympathetic tone [20]. In human studies, the Gly389 allele was under-represented in Japanese acute MI patients relative to controls, and similar findings in heart failure suggested that this variant might be protective [21,22]. However, subsequent cohort studies did not identify any genotype-related differences in adverse cardiovascular outcomes [9,23]. To the contrary, we identified an excess risk of cardiovascular events in patients with the Gly389 allele.

A recent investigation offers compelling mechanistic data to support this unexpected finding of increased risk. Akhter et al. [24] found that hearts from transgenic mice over-expressing cardiac Gly389 showed significantly poorer systolic and diastolic recovery after ischemia-reperfusion compared with Arg389 mice and non-transgenic littermates. Functional data further showed that the Arg389 receptor displayed enhanced phosphorylation, leading to desensitization and increased anti-apoptotic signaling. To the extent that women participating in WISE were enrolled based on chest pain suggestive of ischemia, diminished post-ischemic myocardial recovery associated with the Gly389 allele could be a biologically plausible explanation of our finding that the Gly389 allele increased the risk for heart failure, more so among patients without obstructive CAD. The role of the polymorphism in the pathogenesis of myocardial infarction is less clear, although it is possible that less severe ischemic episodes may actually result in myocardial damage in certain patients. Interestingly, the association was primarily seen in women without obstructive lesions, in whom collateral development and ischemic preconditioning are less likely to compensate. Thus, the outlined mechanisms involving apoptotic signaling may be particularly relevant in this subset of women, whereas other factors may be pervasive in women with flow limiting stenosis.

Among women without obstructive CAD, we also identified an association between composite outcome and the Trp64Arg variant in the β_3_-adrenergic receptor, which has been associated with reduced agonist-stimulated adenylyl cyclase activity *in vitro *[25]. Higashi, et al. [26] reported a higher frequency of the variant among Japanese patients with CAD, although this was not substantiated by subsequent investigations [27-29]. Considering the previously reported associations between the Trp64Arg polymorphism and pro-atherogenic metabolic diseases such as diabetes, we expected that the variant might be indirectly associated with cardiovascular risk [30]. However, the Trp64Arg polymorphism was not associated with BMI, waist circumference, or diabetes in the WISE population [31]. Moreover, the association with the primary outcome was robust to adjustment for these factors.

Beyond metabolic functions, β_3_-adrenergic receptors regulate cardiac inotropy, angiogenesis, and endothelium-dependent vasorelaxation in the coronary microvasculature [32,33]. WISE data demonstrated that women without obstructive CAD have evidence of ischemia using the gold standard cardiac magnetic resonance spectroscopy [4], and that this ischemia is associated with an adverse prognosis [6]. Panting, et al. [3] further demonstrated that subendocardial hypoperfusion may drive myocardial ischemia in patients who have typical angina or abnormal stress test results but no angiographic evidence of severe coronary arterystenosis. Recent WISE data suggest that this may be due to microvascular dysfunction.[34] Thus, compromised β_3_-adrenergic receptor signaling could ostensibly promote ischemia in the microvasculature. While the association with clinical outcomes in WISE was modest, our results suggest that this receptor may play an important role in patients with ischemia in the absence of obstructive CAD and warrant further investigation.

Logic regression is a novel and very powerful approach to defining high-level gene-gene or gene-environment interactions. As the SNPs included in this investigation spanned a biological pathway, we examined whether the genetic variations interacted with each other to modify cardiovascular risk. Previously, this had been demonstrated for renin-angiotensin system polymorphisms in the Group Health Cooperative of Puget Sound [17]. We modeled complex interactions between all of the SNPs, although the best fitting model was actually based on the single SNP in *ADRB1 *that was identified in the initial analyses. These results highlight that single SNP associations remain informative, although gene-gene interactions within biological systems should not be ignored in the setting of complex disease.

WISE was a prospective, multicenter cohort study with a long follow-up period, although several limitations deserve consideration. First, the population may not be large enough to detect subtle genetic or epistatic influences on cardiovascular risk, particularly for the individual outcomes and patient subgroups. As an exploratory investigation, analyses were not adjusted for multiple comparisons, although statistical significance would not have been met using the stringent Bonferonni correction. Mechanistic or replication studies are therefore necessary to make causal inferences for this patient population. While independent replication is the most desirable approach, the WISE cohort represents a unique population of women with detailed angiography whose cardiac disease is largely driven by microvascular defects. Thus, the existing genetic databases (e.g. Framingham, Women's Health Initiative, Wellcome Trust Case-Control Consortium) may not adequately represent the women enrolled in this investigation. Secondly, outcomes were largely ascertained by patient report, although events were adjudicated by the WISE Steering Committee when such data were available. Thirdly, while potentially interesting, genetic associations were not tested relative to β-blocker therapy because data on new use and discontinuation throughout the study period was not sufficient to justify such comparisons. Fourthly, it is interesting that typical risk factors such as hypertension and dyslipidemia were not associated with outcomes. This may be a reflection of active treatment, considering that the mean blood pressure of hypertensive women at entry was 143/79. Lastly, the results may not be generalizable beyond women with ischemic symptoms.

## Conclusion

This investigation demonstrated that SNPs in the genes encoding the β_1_- and β_3_-adrenergic receptors may influence the risk for cardiovascular events among U.S. women with suspected CAD. More importantly, the association in the subset of women without obstructive lesions provides mechanistic insight into the pathophysiology of myocardial ischemia in this population and the relative importance of certain receptor subtypes in vascular function. If replicated, these findings may have significant implications for assessing cardiovascular risk in women without angiographic evidence of severe stenosis, a population for whom risk stratification has been clinically challenging. However, translating these findings to clinical practice will require validation in larger populations, as well as studies to determine the appropriate management strategies for patients with different genetic characteristics.

## Authors' contributions

MP was involved with analysis and interpretation of data, and drafting of the manuscript. IZ was involved with the conception and design of study or analysis and interpretation of data, and drafting of the manuscript. HL was involved with analysis and interpretation of data, and revising manuscript critically for important intellectual content. RMCD, DJ, VB, DM, BS, and CNBM were involved with interpretation of data and revising manuscript critically for important intellectual content. CP was involved with the conception and design of the study and analysis and interpretation of data, manuscript or revising it critically for important intellectual content JJ was involved with the conception and design of the study and analysis and interpretation of data, drafting of the manuscript, manuscript or revising it critically for important intellectual content. All authors read and approved the final manuscript.
